# ADAM12 is A Potential Therapeutic Target Regulated by Hypomethylation in Triple-Negative Breast Cancer

**DOI:** 10.3390/ijms21030903

**Published:** 2020-01-30

**Authors:** Saioa Mendaza, Ane Ulazia-Garmendia, Iñaki Monreal-Santesteban, Alicia Córdoba, Yerani Ruiz de Azúa, Begoña Aguiar, Raquel Beloqui, Pedro Armendáriz, Marta Arriola, Esperanza Martín-Sánchez, David Guerrero-Setas

**Affiliations:** 1 Molecular Pathology of Cancer Group, Navarrabiomed, ComplejoHospitalario de Navarra (CHN), Universidad Pública de Navarra (UPNA), Instituto de Investigación Sanitaria de Navarra (IdiSNA), Irunlarrea 3, 31008 Pamplona, Spain; smendazl@navarra.es (S.M.); ane.ulazia.garmendia@gmail.com (A.U.-G.); dguerres@navarra.es (D.G.-S.); 2 Department of Pathology, ComplejoHospitalario de Navarra (CHN), Irunlarrea 3, 31008, Pamplona, Spain; alicia.cordoba.iturriagagoitia@navarra.es (A.C.); yerani.ruizdeazua.ciria@navarra.es (Y.R.d.A.); begona.aguiar.losada@navarra.es (B.A.); raquel.beloki.perezdeobanos@navarra.es (R.B.); marta.arriola.oses@navarra.es (M.A.); 3 Department of Surgery, ComplejoHospitalario de Navarra, (CHN), Irunlarrea 3, 31008, Pamplona, Spain; pedro.armendariz.rubio@navarra.es

**Keywords:** ADAM12, DNA methylation, triple-negative breast cancer, epigenetic biomarkers, therapeutic target

## Abstract

Triple-negative breast cancer (TNBC) is the most aggressive breast cancer subtype and currently lacks any effective targeted therapy. Since epigenetic alterations are a common event in TNBC, DNA methylation profiling can be useful for identifying potential biomarkers and therapeutic targets. Here, genome-wide DNA methylation from eight TNBC and six non-neoplastic tissues was analysed using Illumina Human Methylation 450K BeadChip. Results were validated by pyrosequencing in an independent cohort of 50 TNBC and 24 non-neoplastic samples, where protein expression was also assessed by immunohistochemistry. The functional role of disintegrin and metalloproteinase domain-containing protein 12(*ADAM12*) in TNBC cell proliferation, migration and drug response was analysed by gene expression silencing with short hairpin RNA. Three genes (Von Willenbrand factor C and Epidermal Growth Factor domain-containing protein *(VWCE)*, tetraspanin-9 (*TSPAN9*) and *ADAM12*) were found to be exclusively hypomethylated in TNBC. Furthermore, *ADAM12* hypomethylation was associated with a worse outcome in TNBC tissues and was also found in adjacent-to-tumour tissue and, preliminarily, in plasma from TNBC patients. In addition, *ADAM12* silencing decreased TNBC cell proliferation and migration and improved doxorubicin sensitivity in TNBC cells. Our results indicate that ADAM12 is a potential therapeutic target and its hypomethylation could be a poor outcome biomarker in TNBC.

## 1. Introduction

Breast cancer (BC) is the most common tumour type in women worldwide and the leading cause of cancer-related deaths for females, with an estimated 2.1 million new cases in 2018 [1]. BC is a highly heterogeneous disease categorised into several molecular subtypes with a variety of biological features and clinical outcomes. This classification is based on the differential expression, detected by microarrays, of crucial genes in cancer onset and progression [2]. However, due to logistic and economic constraints, surrogate approaches have been developed for routine clinical practice, using widely available immunohistochemistry (IHC) assays for oestrogen receptor (ER), progesterone receptor (PR) and the Ki-67 index, along with IHC and/or in situ hybridization for human epidermal growth factor 2 receptor (HER2) [3].

Taking into account the IHC expression of these biomarkers, the classification adopted in the thirteenth St Gallen International Breast Cancer Conference in 2013 [4] divides BC into five molecular subtypes: Luminal A, Luminal B/HER2-negative, Luminal B/HER2-positive, HER2-positive and triple-negative BC (TNBC). Of these, TNBC accounts for 10–20% of all diagnosed BCs, and is the most aggressive subgroup, characterised by early relapse, frequent distant metastasis and poor overall survival [2,5]. TNBC lacks expression of receptors that are therapeutically useful in other subtypes, and there is currently no targeted treatment available for these patients. TNBC therapy is therefore a serious clinical challenge [6] and the identification and evaluation of new biomarkers and therapeutic targets is a high priority in TNBC research.

DNA methylation is the most well-known epigenetic modification in human disease and has been implicated in regulating the expression of a great variety of genes that are critical in cancer [7]. DNA methylation status has emerged as one of the most promising epigenetic biomarkers for several types of cancer, including BC [7,8], since it can be used in early detection and prediction of prognosis or response to treatment [9,10]. For example, O-6-Methylguanine-DNA Methyltransferase (MGMT) methylation is currently used by clinicians for routine evaluation of glioma patients’ therapeutic response to temozolomide [11]. However, few aberrantly methylated genes have been reported in TNBC, and none of them have yet been implemented in clinical practice. In this context, the discovery of key molecular alterations and the understanding of their functional consequences would allow us to propose new biomarkers of clinical utility in TNBC. Therefore, our aim was to identify new aberrantly methylated genes of clinical value and to understand their biological role in TNBC.

## 2. Results

### 2.1. Genome-Wide DNA Methylation Pattern in TNBC Patients

**Figure 1:**
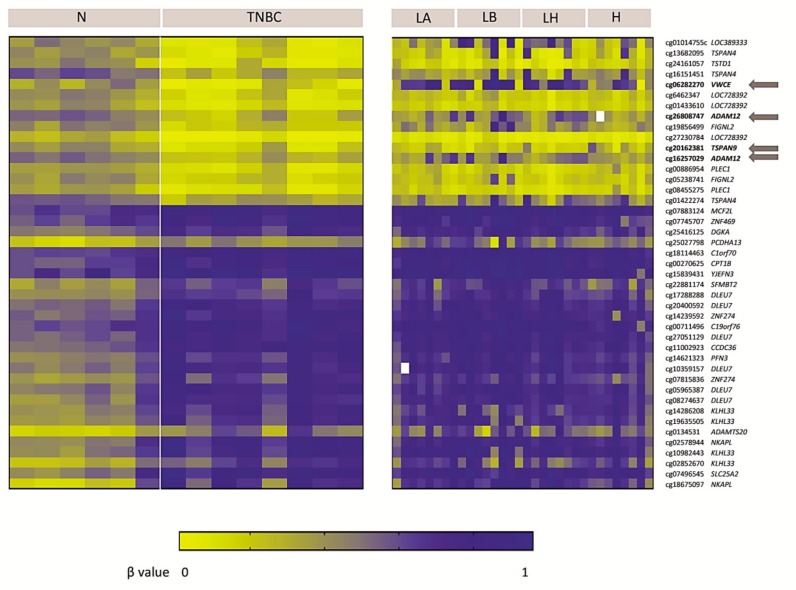
DNA methylome of triple-negative breast cancer (TNBC). Heat-map showing differentially methylated probes in the 5′UTR region, at 1500–200 bp from the transcription start site or in the first exon (False Discovery Rate (FDR) < 0.05; Δβ > 0.2) and their corresponding genes in TNBC tissues compared with non-neoplastic breast tissues (N), and other BC subtypes (Luminal A (LA), Luminal B/human epidermal growth factor 2 receptor (HER2)-negative (LB), Luminal B/HER2-positive (LH), and HER2-positive (H)). Genes with altered methylation exclusively in TNBC, but not in other BC subtypes, are highlighted with an arrow.

The DNA methylome of a small series of eight TNBCs was compared with that of six non-neoplastic breast tissues using a methylation array. We found 43 significally differentially methylated probes (DMPs)(False Discovery Change(FDR) < 0.05), with a Δβ> 0.2, and located within CpG islands in the 5′UTR region, 1500–200 bp upstream of the transcription start site or within the first exon. In particular, we found 27 and 16 probes, which recognised 17 and 10 hypermethylated and hypomethylated genes respectively, in TNBC relative to non-neoplastic tissue (Figure 1). We then examined whether this methylation pattern was exclusive to the TNBC subtype or common to other BC subtypes by interrogating the TNBC methylation signature in a series of Luminal A, Luminal B/HER2-negative, Luminal B/HER2-positive and HER2-positive BC patients (eight per group). Only four probes recognising three genes (Von Willenbrand factor C and Epidermal Growth Factor domain-containing protein *(VWCE)*, tetraspanin-9 (*TSPAN9)* and disintegrin and metalloproteinase domain-containing protein 12 (*ADAM12*)) were found to be exclusively hypomethylated in the TNBC subtype. They remained hypermethylated or not significantly altered in the other subtypes (Figure 1). These results suggest that TNBC has a different DNA methylation pattern from that of non-neoplastic breast tissue, and that hypomethylation of particular genes is exclusive to the TNBC subtype.

### 2.2. VWCE, TSPAN9 and ADAM12 Methylation Levels Are Lower in TNBCs Than in Non-Neoplastic Breast Tissues 

**Figure 2:**
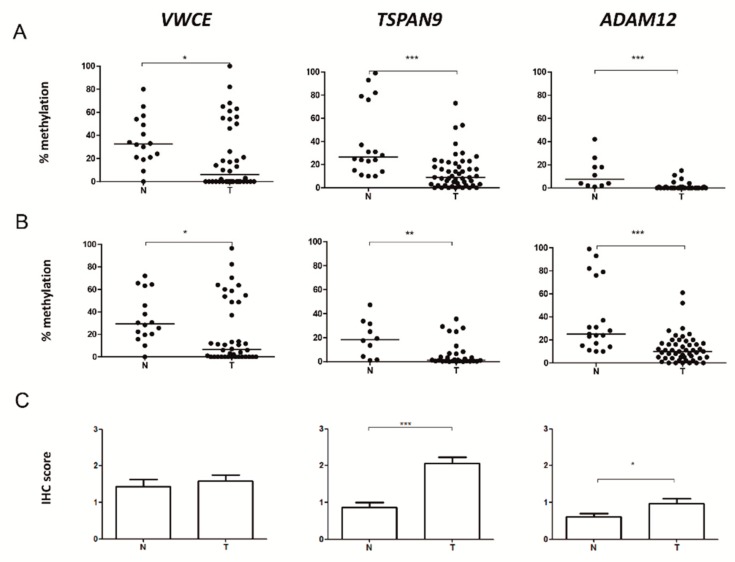
Methylation and protein levels of Von Willenbrand factor C and Epidermal Growth Factor domain-containing protein (*VWCE*), tetraspanin-9 (*TSPAN9*) and disintegrin and metalloproteinase domain-containing protein 12 (*ADAM12*) genes in breast tissues. (**A**) Methylation percentage of the CpG included in the array and (**B**) the mean of all the analysed CpGs in each gene exclusively hypomethylated in TNBC were measured by pyrosequencing in non-neoplastic breast (N) and TNBC (T) tissues. The horizontal lines represent the median of the series. (**C**) Levels of proteins encoded by those genes was determined by immunohistochemistry (IHC) in non-neoplastic samples (N) and TNBC (T). Expression was scored as: 0, no expression; 1, weak expression; 2, intermediate expression; and 3, strong expression. (*, *p* < 0.05; **, *p* < 0.01; ***, *p* < 0.001).

To validate data derived from the DNA methylation array, we focused on the only three genes carrying specific aberrant methylation in TNBC but not in other BC subtypes: *VWCE, TSPAN9* and *ADAM12*. For each gene, the methylation status of a region covering the DMP in the array and some contiguous CpG sites was analysed by pyrosequencing in a larger series of 50 TNBCs and 24 non-neoplastic breast tissues. We confirmed that TNBC tumours had significantly lower methylation levels than non-neoplastic samples in all analysed CpGs in *VWCE*, *TSPAN9* and *ADAM12* (*p* < 0.05) (Supplementary Table S1). Methylation of the CpG included in the array is illustrated in Figure 2A, and the mean methylation levels of all analysed CpGs are shown in Figure 2B.

### 2.3. Level of Expression of TSPAN9 and ADAM12 is Higher in TNBCs Than in Non-Neoplastic Breast Tissue

**Figure 3:**
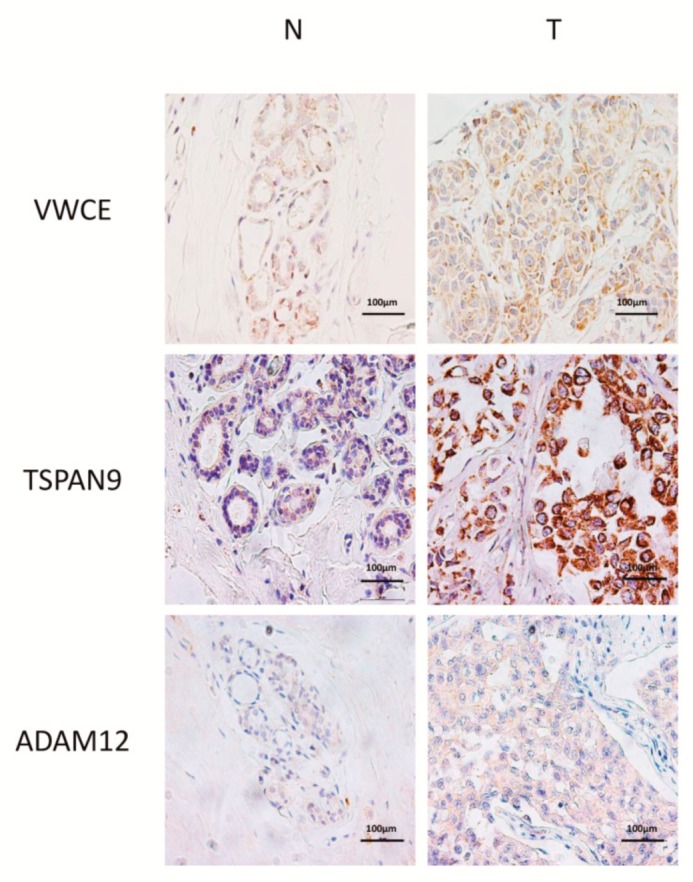
Representative IHC of non-neoplastic (N) and triple-negative breast cancer (T) tissues of VWCE, TSPAN9 and ADAM12 proteins. Images were acquired at 400× magnification.

To explore whether *VWCE, TSPAN9* and *ADAM12* hypomethylation affected protein expression, IHC was performed in 25 TNBCs and 24 non-neoplastic breast tissue samples. We observed that TSPAN9 and ADAM12, but not VWCE, protein levels were significantly higher in tumours than in non-neoplastic tissues (*p* < 0.05) (Figure 2C, Figure 3 and Figure S1). These findings indicate that TNBC tissues with hypomethylated *TSPAN9* and *ADAM12* genes also exhibit overexpression of TSPAN9 and ADAM12 proteins relative to non-neoplastic breast tissue. 

### 2.4. Adjacent Non-Neoplastic Tissue Has a DNA Methylation Pattern Similar to that of TNBCs but Different from that of Non-Neoplastic Mammary Tissue

**Figure 4:**
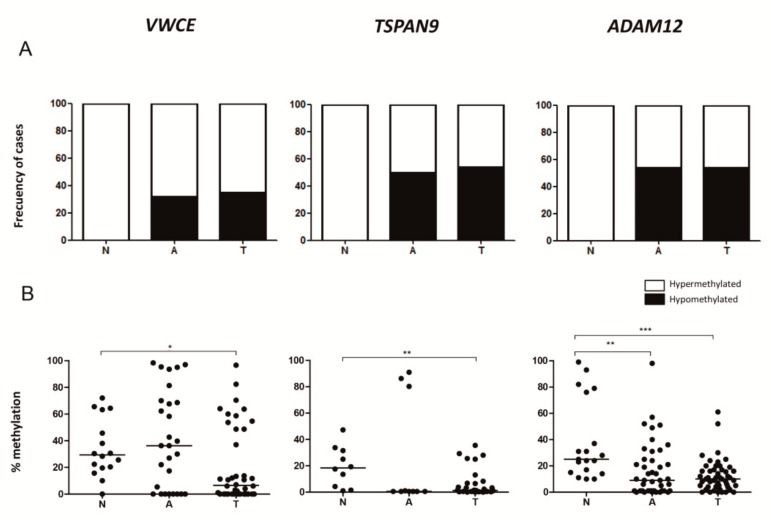
Methylation status of *VWCE*, *TSPAN9* and *ADAM12* genes in breast tissues. (**A**) Percentages of hypomethylated and hypermethylated cases are represented. Samples with methylation levels below the minimum percentage of methylation observed in our non-neoplastic tissue series are considered hypomethylated cases. (**B**) Mean methylation percentage of all the analysed CpGs in each gene was measured by pyrosequencing in non-neoplastic breast (N), adjacent-to-tumour (A) and TNBC (T) tissues. The horizontal lines represent the median of the series (*, *p* < 0.05; **, *p* < 0.01; ***, *p* < 0.001).

We further analysed the methylation status of *VWCE, TSPAN9* and *ADAM12* genes in 45 adjacent-to-tumour tissues. The proportion of hypomethylated cases was significantly higher in adjacent-to-tumour tissues than in non-neoplastic tissues in all genes (*p*< 0.05), but similar to that of the TNBC samples (Figure 4A). We also observed that adjacent-to-tumour samples, without apparent neoplastic cell morphology, harboured a significant loss of *ADAM12* methylation compared with non-neoplastic cases (*p*< 0.05) (Figure 4B). These results indicate that some epigenetic alterations commonly found in TNBC could already be present in the adjacent-to-tumour but non-neoplastic tissue, suggesting that they may be involved in the cell transformation process.

### 2.5. Clinical Value of ADAM12 Hypomethylation in TNBC

**Figure 5:**
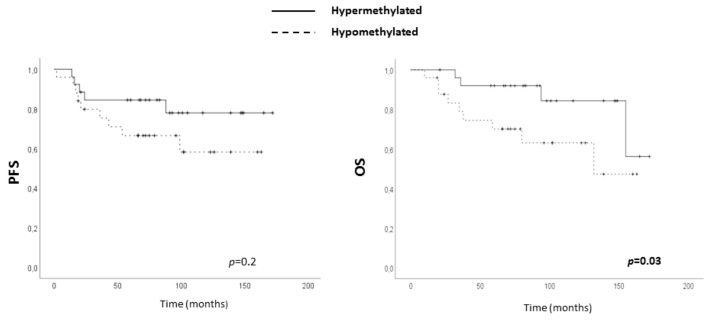
Clinical value of *ADAM12* hypomethylation in TNBC. Association between *ADAM12* hypomethylation and progression-free survival (PFS) (left panel) and overall survival (OS) (right panel) in our series of TNBC patients.

Since we had found aberrant DNA methylation in TNBC, the clinical importance of *ADAM12*, *TSPAN9* and *VWCE* hypomethylation was assessed in our series of 50 TNBC patients. Pyrosequencing provides a quantitative measure of methylation, so a cut-off value distinguishing between hypomethylated and hypermethylated status was established for each gene using the minimum percentage of methylation observed in our non-neoplastic breast series: 0% for *VWCE*, 1% for *TSPAN9* and 10% for *ADAM12*. On this basis, no association between any tested hypomethylation and progression-free survival (PFS) was found. However, hypomethylation of *ADAM12*, but not of *TSPAN9* and *VWCE*, had a significant impact on OS (Figure 5 and Figure S2), although its independence from well-established clinical parameters, age and stage, was not statistically significant (Supplementary Table S2). Thus, association of *ADAM12* hypomethylation and those relevant parameters was assessed but they did not show statistical association (age (*p* = 0.80) and stage (*p* = 0.18)).

### 2.6. ADAM12 Silencing Inhibits TNBC Cell Proliferation and Migration

**Figure 6:**
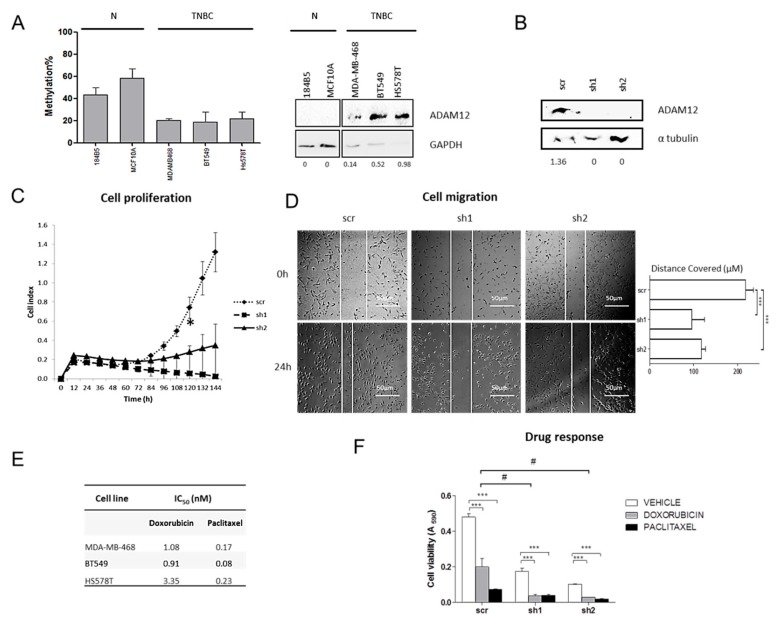
Effects of *ADAM12* silencing on TNBC cell lines. (**A**) *ADAM12* methylation (left panel) and protein (right panel) levels were assessed by pyrosequencing and western blot respectively, in a panel of two non-neoplastic mammary cells (N) and three TNBC cell lines. Numbers indicate the amount of ADAM12 relative to that of GAPDH, as measured by densitometry. (**B**) In order to silence *ADAM12* expression, BT-549 cells were transfected with pHIV1-SIREN + scramble (scr), pHIV1-SIREN + shADAM12_1 (sh1), and pHIV1-SIREN + shADAM12_2 (sh2). After selection of transfected cells with puromycin, *ADAM12* depletion efficiency was checked by western blot in two independent experiments. Numbers indicate the mean amount of ADAM12 relative to that of α-tubulin. (**C**) Stable transfected and therefore, *ADAM12*-silenced-BT-549 cell proliferation was measured by real-time cell analysis for 6 days. (**D**) Effects of *ADAM12* knockdown on BT-549 cell migration were measured for 24 h. Images were acquired at 50× magnification. The distance covered by cells (μm) over 24 h is also shown in the histogram. (**E**) Specific IC_50_ values for each cell lline and drug. (**F**) Effects of *ADAM12* silencing on BT-549 response to doxorubicin and paclitaxel were assessed by measuring cell viability upon *ADAM12* knockdown and doxorubicin treatment. (*, *p* < 0.05; **, *p* < 0.01; ***, *p* < 0.001) (#, *p* < 0.05).

To determine the biological role of *ADAM12* in TNBC, we first assessed its methylation and expression status in a panel of three TNBC cell lines and two non-neoplastic but immortalised mammary cell lines. Similar to the tissues, *ADAM12* in TNBC cells was hypomethylated and overexpressed relative to non-neoplastic breast cells (Figure 6A), indicating that these cell lines were tissue representative. Then, we inhibited *ADAM12* expression in two TNBC-derived cell lines with low levels of methylation and the highest protein levels of ADAM12 (BT-549 and Hs 578T), using two short hairpin RNAs (shRNAs) against *ADAM12*. Western blot revealed that shADAM12_1 and shADAM12_2 both entirely depleted ADAM12 protein in BT-549 cells (Figure 6B). Under these conditions, both types of shADAM12 significantly decreased BT-549 cell proliferation after 120 h (Figure 6C), and cell migration (Figure 6D) in comparison with the scramble (*p* < 0.05). No molecular and functional assays could be performed in shADAM12-transfected Hs 578T cells because they did not survive, but scramble-transfected cells did (Supplementary Figure S3). These observations indicate that ADAM12 overexpression caused, at least in part, by hypomethylation, could promote TNBC cell aggressiveness. Therefore, we conclude that ADAM12 is a potential therapeutic target in TNBC.

### 2.7. ADAM12 Silencing Improves Doxorubicin Sensitivity of TNBC Cells

To investigate whether ADAM12 was involved in the response to chemotherapeutic agents commonly administered to TNBC patients, such as doxorubicin and paclitaxel, their IC_50_ values were calculated in three TNBC cell lines. It is of particular note that the cell line with the strongest ADAM12 expression, Hs 578T (Figure 6A), had the highest IC_50_ value for both doxorubicin and paclitaxel, while MDA-MB-468 and BT-549, with weaker ADAM12 expression, had lower IC_50_values (Figure 6E). This observation suggests that ADAM12 may be associated with drug response in TNBC. Therefore, *ADAM12*-silenced-BT-549 cells were treated with doxorubicin or paclitaxel for 72 h (Figure 6F). *ADAM12* silencing effects on cell proliferation were also noted (similar to Figure 6C). We observed that *ADAM12* inhibition significantly reduced cell viability to a similar extent as did doxorubicin in scramble-transfected cells. Additionally, while paclitaxel treatment did not significantly differently affect shADAM12-transfected cell viability compared with scramble-transfected ones (data not shown), doxorubicin dramatically decreased *ADAM12*-silenced-BT-549 cell viability (Figure 6E). These findings indicate that ADAM12 plays an important role in doxorubicin resistance in TNBC.

### 2.8. ADAM12 Is Hypomethylated in Plasma from TNBC Patients

**Figure 7:**
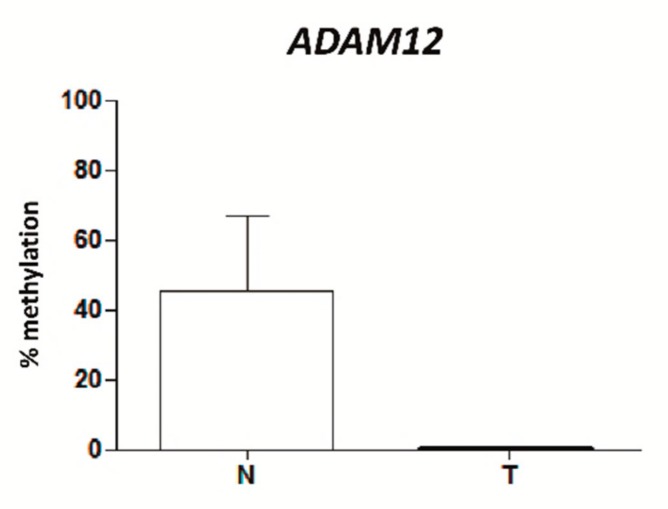
Percentage methylation of the mean of all the analysed CpGs in the *ADAM12* gene in TNBC was measured by pyrosequencing in non-neoplastic breast (N) and TNBC (T) total circulating cell-free DNA (cfDNA) from plasma.

Given the importance of *ADAM12* hypomethylation in the molecular and clinical pathology of TNBC, we examined whether this epigenetic alteration could also be detected by non-invasive methods. To this end, the levels of *ADAM12* methylation in total cell-free (cfDNA) were studied in a small series of plasma from six TNBC patients and 13 healthy women. All TNBC patients lacked *ADAM12* methylation, while healthy women harboured around 40% of *ADAM12* methylation (Figure 7). Additionally, *ADAM12* methylation was also tested in formalin-fixed, paraffin-embedded (FFPE (FFPE) tumours from these TNBC patients. All FFPE TNBC tumours showed 0% *ADAM12* methylation, as their matched cfDNA (data not shown). Although the sample size was very small (*n* = 3), these data raise the possibility that *ADAM12* is hypomethylated, relative to that of healthy women, not only in the tumour tissue, but also in the cfDNA released into the plasma from TNBC patients. Its highly representative nature and the ease by which it can be extracted by non-invasive methods suggest that cfDNA may be an appropriate material in which to test relevant epigenetic biomarkers in TNBC patients.

## 3. Discussion

TNBC is associated with poor long-term outcomes compared with other BC subtypes [12]. Despite current research focused on understanding the molecular landscape of TNBC, reliable prognostic and predictive biomarkers and targeted therapies remain lacking from clinical practice [13]. Some genetic biomarkers have been proposed in TNBC [14], but few methylation studies have been carried out in this specific BC subtype. To date, only four studies have analysed whole-genome DNA methylation in TNBC. Two of these attempted to shed some light on TNBC subclassification by characterising its DNA methylome [15,16], since TNBC is a heterogeneous group defined by the lack, not the presence, of certain biomarkers. Conversely, the other two studies addressed TNBC biological mechanisms in greater depth by identifying driver molecular alterations in the DNA methylome in comparison with non-neoplastic breast samples [17,18], using adjacent-to-tumour tissues as non-neoplastic controls. However, it has been widely reported that tissues surrounding tumours frequently appear histologically normal but show pre-neoplastic molecular alterations [19,20,21,22,23]. This phenomenon is known as the “field effect” [19]. In particular, we [24] and others [25,26] have described that adjacent-to-tumour breast tissue contains changes in DNA methylation that may contribute to tumour initiation, and thereby possibly be markers of the onset of neoplasia. Accordingly, our results demonstrate that the field effect could also happen in TNBC, since hypomethylation of one of the three selected genes is already present in the adjacent-to-tumour but morphologically non-neoplastic breast tissue. This effect might have biased the results found by other authors [17,18], who compared the TNBC with adjacent tissue, instead of purely non-neoplastic tissue. To avoid this discrepancy, our study used non-neoplastic samples from reduction mammoplasties as controls. We found that TNBC has a different DNA methylation pattern compared with purely non-neoplastic breast tissue, and we identified three novel genes (*VWCE*, *TSPAN9* and *ADAM12*) that were hypomethylated in TNBC but not in other BC subtypes. To the best of our knowledge, their methylation status has not been described in any other cancer type.

First, the *VWCE* (Von Willenbrand factor C and Epidermal Growth Factor domain-containing protein) gene encodes a protein that is overexpressed in many cancer tissues and cell lines, and that promotes cancer development and progression [27]. However, the mechanism responsible for its upregulation has not been elucidated. Here, we reported for the first time an aberrant hypomethylation of *VWCE* in cancer, which would explain the overexpression described in the literature [27]. 

Second, *TSPAN9* (tetraspanin-9) belongs to a protein superfamily that is involved in cell development, differentiation, mobility, as well as in tumour proliferation and invasion. In particular, the role of *TSPAN9* in cancer has not been thoroughly explored: a lower level of expression in gastric cancer than in non-neoplastic gastric tissue [28], and some anti-tumour effects in vitro gastric models [29,30], are the only findings reported so far. In contrast, here, we described a higher level of expression of TSPAN9 in TNBC than in non-neoplastic counterparts, suggesting that *TSPAN9* might have a tumour-dependent molecular status and role. Moreover, our results provide a plausible explanation for TSPAN9 deregulation in cancer, as we demonstrated that aberrant *TSPAN9* methylation could regulate its expression in TNBC. 

Finally, the third gene found to be abnormally methylated in TNBC is *ADAM12* (disintegrin and metalloproteinase domain-containing protein 12). It belongs to a matrix metalloproteinase-related protein family and participates in the proteolytic processing of other transmembrane proteins, with consequences for cell-signalling events, transcription, RNA metabolism, apoptosis, cell-cycle progression, and cell adhesion. ADAM12 overexpression has been reported in many tumours [31,32], especially in BC, where it has been proposed to make an important contribution in carcinogenesis [33,34,35,36]. However, its molecular status in the TNBC subtype is almost entirely unexplored. In this study, we demonstrated ADAM12 overexpression in TNBC tissues and cell lines. Accordingly, a recent study has reported a higher level of expression in the claudin-low subset of TNBC compared with that in other BC subtypes, at the mRNA level in tissues and the protein level in cell lines [37]. As is the case of the proteins described above, the mechanism underlying ADAM12 upregulation has not yet been elucidated. Since we have also demonstrated its lower methylation level in TNBC tissues and cell lines, we propose that ADAM12 overexpression in TNBC could be mediated, at least in part, by DNA hypomethylation. More importantly, we demonstrated for the first time that this epigenetic alteration has a significant impact on the OS of TNBC patients. These findings are consistent with the reported association between high levels of ADAM12 expression and poor prognosis in TNBC, but not in the rest of BC subtypes [38].

Since we found DNA to be hypomethylated in tumour and adjacent-to-tumour tissue relative to non-neoplastic samples, leading to protein overexpression and worse OS in TNBC, our results suggested a potential key role for ADAM12 in TNBC, which prompted us to investigate its biological function in TNBC. Here we demonstrate that *ADAM12* silencing inhibits TNBC cell proliferation and migration in vitro, a finding that is consistent with those of the only study showing tumour-initiation and growth effects of *ADAM12* silencing in a TNBC in vivo model [37]. Besides tumour growth and metastasis, it is interesting to note that some of the ADAM family members play important roles in chemoresistance and recurrence of tumours [39]. Accordingly, several studies have shown ADAM12 mRNA overexpression in chemo-resistant ER-negative breast tumours [40,41]. In addition, ADAM12 re-expression in the non-malignant breast epithelial MCF 10A cell line has been reported to induce resistance to cisplatin [42], while *ADAM12* silencing facilitates 5-fluorouacil sensitivity in a TNBC xenograft model [39]. Consistent with these findings, paclitaxel administration has been shown to increase ADAM12 protein levels in the SUM159PT TNBC cell line [37]. These observations suggested that ADAM12 could be mechanistically involved in chemoresistance in TNBC, which is one of the main causes of recurrence and aggressiveness in these patients [43]. In this context, we have explored the sensitivity of *ADAM12*-silenced TNBC cells to doxorubicin and paclitaxel, as models of anthracycline and taxane-based chemotherapy, the standard of care for TNBC [44]. We demonstrated that simultaneous *ADAM12* silencing and doxorubicin treatment dramatically decreased BT-549 cell viability. A similar trend has been described in *ADAM12*-silenced MDA-MB-231 cells, although the results were not statistically significant, probably due to the incomplete knockdown of A*DAM12* [39]. Therefore, based on this, ADAM12 can be proposed as a potential therapeutic target for TNBC patients. Further studies in TNBC in vivo models will address the therapeutic improvement of doxorubicin by ADAM12 inhibition.

In recent years, non-invasive methods of biomarker identification, such as liquid biopsy, have been attracting increasing interest in cancer research [45,46]. Liquid biopsy includes isolation of cfDNA, which can be detected in the plasma of cancer patients even during the early stages of their disease [47]. Furthermore, cfDNA from cancer patients is known to carry tumour-specific changes in DNA methylation that are not present in the cfDNA of healthy donors [48,49]. Based on this, panels of tumour-specific methylated genes of potential value for early detection of BC have been described in cfDNA [50,51], including the *RASSF1A*, *PITX2* [52,53] and *EFC* [54] genes, whose hypermethylation has been associated with poor prognosis of BC. Despite these promising findings, epigenetic alterations in cfDNA have not so far been explored in TNBC. For instance, our discovery of *ADAM12* hypomethylation in tumour and in cfDNA from TNBC patients, although in a very small series, would support the proof of concept to carry out these analyses in larger cohorts. Future analysis also will need to assess *ADAM12* methylation status in haematopoietic cells as intrasample control since, besides tumour, those cells are also a source of cfDNA. This would allow us to exclude that the methylation alteration found in plasma is a patient variation itself and to establish beyond doubt the usefulness of cfDNA as an informative material for biomarker identification in TNBC. 

To summarise, here we report for the first time that: (i) *ADAM12* is hypomethylated and overexpressed in TNBC cases relative to non-neoplastic breast tissues, (ii) this epigenetic alteration is already present in the adjacent-to-tumour tissue and in cfDNA, (iii) ADAM12 promotes TNBC cell proliferation, migration and doxorubicin-resistance, and (iv) low levels of *ADAM12* methylation are associated with shorter OS in TNBC patients. We conclude that ADAM12 is a potential therapeutic target and its hypomethylation could be a biomarker of poor outcome in TNBC. 

## 4. Materials and Methods 

### 4.1. Patient Samples

Three patient series were used in this study. First, an initial series of frozen tissues from eight TNBCs and six non-neoplastic breast tissues from reduction mammoplasties was used to characterise the TNBC methylome. Additionally, 32 frozen BC samples were used to identify and thereby discount similarities in the DNA methylation pattern with other BC subtypes. Then, a second series of FFPE samples, consisting of 50 TNBCs, 45 matched non-neoplastic but adjacent-to-tumour tissues and 24 non-neoplastic breast tissues from reduction mammoplasties, was employed to validate the results of the methylome analysis and to assess the protein expression. Finally, a small series of plasma samples from 6 TNBC patients and 13 healthy women of matched age was used to explore the methylation status of selected genes in total cell-free DNA (cfDNA). All patients were diagnosed with infiltrating duct breast carcinoma in the Department of Pathology (Complejo Hospitalario de Navarra, Pamplona, Spain) in accordance with the criteria recommended by the St Gallen International Expert Consensus 2013 [4], considering a specific Ki-67 threshold [54], grading according to the Nottingham system [55] and staging based on the American Joint Committee on Cancer (AJCC) system [56]. It was ensured that all cancer samples harboured at least 70% tumour cells. None of the patients had received radiotherapy or chemotherapy before surgery. Their pathological and clinical characteristics are summarised in Supplementary Table S3.

### 4.2. Cell Lines

A panel of three human TNBC cell lines was used in this study: BT-549, which was purchased from the American Type Cell Collection (ATCC, Rockville, MD, USA), MDA-MB-468, which was obtained from the German Collection of Microorganisms and Cell Cultures (DSMZ, Braunschweig, Germany), and Hs 578T, which was kindly provided by Dr Javier Benítez (Human Genetics Group, Spanish National Cancer Research Centre, Madrid, Spain). All TNBC cell lines were grown in RPMI-1640 or DMEM, supplemented with 10% foetal bovine serum (FBS) and 1% penicillin/streptomycin (all from Lonza Biologics, Basel, Switzerland), at 37 °C in a humidified atmosphere with 5% CO_2_. Two immortalised but non-tumorigenic human mammary cell lines were also used: 184B5 cells were obtained from the ATCC (Rockville, MD, USA), and the MCF 10A cell line was kindly provided by Dr Green (Molecular, Cell and Cancer Biology Department, University of Massachusetts Medical School, Worcester, MA, USA). These non-tumorigenic cell lines were cultured in mammary epithelial basal medium (MEBM) supplemented with 5% horse serum, 10 μg/mL insulin, 0.5 μg/mL hydrocortisone, 20 ng/mL epithelial growth factor, 10% FBS, 1% penicillin/streptomycin (all from Lonza Biologics, Basel, Switzerland) and 100 ng/mL cholera toxin (Sigma-Aldrich, St Louis, MO, USA). All cell lines were *Mycoplasma*-free and authenticated by short tandem repeat (STR) analysis in March 2019.

### 4.3. DNA and Cell-Free DNA (cfDNA) Extraction and Bisulphite Conversion

To analyse DNA methylation status, DNA and total cfDNA was extracted from BC patients’ and healthy women’s tissue (frozen or FFPE) and plasma samples, as well as from cell lines, using the QIAamp DNA FFPE Tissue kit and the QIAamp Circulating Nucleic Acid Kit (both from Qiagen, Hilden, Germany) following the manufacturer’s instructions. 500 ng of DNA or 100 ng cfDNA were bisulphite-converted using the EZ DNA Methylation-Gold kit (Zymo Research, Irvine, CA, USA).

### 4.4. DNA Methylation Array and Bioinformatics Analysis

Bisulphite-converted DNA samples from the initial series of eight TNBCs, six non-neoplastic mammary tissues and 32 BCs were subjected to the Illumina Infinium Methylation 450K BeadChips (Illumina, San Diego, CA, USA) in the Human Genotyping Unit (Spanish National Cancer Research Centre, Madrid, Spain), following the manufacturer’s recommendations. The methylation level of each of the 450,000 CpG sites interrogated in the array was estimated as normalized β values using the GenomeStudio program v2010.3 (Illumina, San Diego, CA, USA). Then, a limma t-test (http://pomelo2.iib.uam.es/) was performed to identify probes that were differentially methylated between tumour and non-neoplastic samples, assuming a false-discovery rate (FDR) of < 0.05. We focused on those DMPs with a value of Δβ (|βtumour – βnon-neoplastic tissue|) > 0.2 and located within a CpG island in the 5′ UTR region, 1500–200 bp upstream of the transcription start site or the first exon of the gene. This location restricted the research to CpG islands whose methylation can regulate gene expression [57]. Raw data from our methylation microarray were deposited in Gene Expression Omnibus (GEO) under accession number GSE141338. 

### 4.5. Pyrosequencing

To validate the differential methylation status of the three selected genes (*VWCE*, *TSPAN9* and *ADAM12*) in TNBC, pyrosequencing was carried out in bisulphite-converted DNA from cell lines, FFPE tissues from our second series of 50 TNBCs, 45 matched adjacent-to-tumour samples and 24 non-neoplastic mammary tissues and cfDNA from the plasmas of the third series of 6 TNBC patients and 13 healthy donors. First, 2 μL of bisulphite-modified DNA were amplified by PCR using 0.5 μL IMMOLASE DNA polymerase (BioLine, London, UK) in a final volume of 30 μL, and with primers that amplified the same region recognised by the probe contained in the array (Supplementary Table S4). Amplification conditions consisted of initial DNA polymerase activation at 95 °C for 10 min, followed by 45 cycles at 95 °C for 30 s, at a specific Tm for each gene (Supplementary Table S4) for 30 s and 72 °C for 30 s, and a final extension at 72 °C for 7 min. Then, pyrosequencing was carried out as previously described [24,58] in a PyroMark Q96 (Qiagen, Hilden, Germany). 

### 4.6. Immunohistochemistry

To measure the protein levels of genes whose differential methylation was validated, IHC was performed in 25 TNBCs and 24 non-neoplastic breast samples. Four-µm thick sections were placed on slides and then deparaffinised, hydrated and treated to block endogenous peroxidase activity. Samples were incubated for 10 min with primary rabbit polyclonal antibodies against VWCE (ab184772, Abcam, Cambridge, UK) at 1: 750, TSPAN9 (J94406, St John’s Laboratory Ltd., London, UK) at 1: 100 and ADAM12 (A7940, ABclonal Technology, Boston, MA, USA) at 1: 100 (antigen retrieval at 90 °C for 20 min, pH = 6.0). Antibodies were then developed using a Bond Polymer Refine Detection kit (Leica, Wetzlar, Germany) and visualised with diaminobenzidine. The expression pattern was evaluated blind by two independent observers. The intensity of expression was ascribed to one of four categories: 0, no expression; 1, weak expression; 2, intermediate expression; 3, strong expression. Images were acquired at 400× magnification with a Leica DM4000B microscope (Leica, Wetzlar, Germany). Validation of the staining of each IHC was carried out using placenta as a positive control tissue, as recommended by the manufacturers.

### 4.7. ADAM12 Silencing in TNBC Cell Lines

To study the functional role of the *ADAM12* gene in TNBC, its expression was silenced by shRNAin BT-549 and Hs 578T cells. For shRNA construction, two sequences targeting *ADAM12* (shADAM12_1:5′-GGCCTGAATCGTCAATGTCAAA-3′ and shADAM12_2: 5′-GCGCTCGAAATTACACGGTAAT-3′), and one scramble sequence (5′-GCAACAAGATGAAGAGCACCAA-3′) were inserted into the pHIV1-SIREN-PuroR plasmid (kindly provided by Dr David Escors, Oncoimmunology Group, Navarrabiomed, Pamplona, Spain) through digestion with *BamHI* and *EcoRI* restriction enzymes (Life Technologies, Carlsbad, CA, USA) and ligation with the T4 DNA ligase enzyme (New England Biolabs, Ipswich, MA, USA). Competent *E. coli* XL1-Bluebacteria were then transformed with these shRNA constructions, plasmids were purified using the Qiagen Plasmid Midi kit (Qiagen, Hilden, Germany) and sequenced to check the ligation. Since the plasmid contained the puromycin-resistance gene for mammalian cell selection, TNBC cell sensitivity to this antibiotic (ThermoFisher, Waltham, MA, USA) was first tested, and a concentration of 1 μg/mL was chosen as optimal from a range of possibilities. BT-549 and Hs 578T cells were then stably transfected with plasmids containing scramble, shADAM12_1 and shADAM12_2, as follows: 5 × 10^4^ cells were seeded in six-well plates, allowed to attach overnight, and then a mixture of 1.2 µg of the plasmid of interest and 1:3 (*v*/*v*) FuGene HD (Promega, Madison, WI, USA) was added in 60 µL of DMEM (Lonza Biologics, Basel, Switzerland, Spain). After 48 h, the culture medium was replaced with fresh medium containing puromycin, and cells were maintained for 2 weeks for selection of transfected cells.

### 4.8. Western Blot

In order to check intrinsic expression of ADAM12 protein and *ADAM12* silencing efficiency in TNBC-derived cell lines, western blots were carried out. Whole-cell protein fraction was extracted using RIPA buffer (Sigma-Aldrich, St Louis, MO, USA) and the complete protease inhibitor cocktail (Roche, Basel, Switzerland) from the three TNBC cell lines (BT-549, Hs 578T and MDA-MB-468), the two immortalised but non-neoplastic mammary cell lines (184B5 and MCF 10A) and the BT-549 cells transfected with scramble, shADAM12_1 and shADAM12_2. After incubating for 5 min on ice and centrifuging at 8000× *g* for 10 min at 4 °C, proteins contained in the supernatants were quantified with the DC protein assay (Bio-Rad Laboratories, Hercules, CA, USA) in an Epoch multi-plate reader (BioTek, Winooski, VT, USA). 60 µg of protein were separated by SDS-PAGE in a 10% polyacrylamide gel and transferred onto a nitrocellulose membrane (Millipore, Billerica, MA, USA), which was blocked with 5% non-fat milk and incubated with the primary rabbit polyclonal anti-ADAM12 (A7940, ABclonal Technology, Boston, MA, USA) at a 1:250 dilution, overnight and at 4 °C. Then, incubation with the secondary anti-rabbit antibody (Bio-Rad Laboratories, Hercules, CA, USA) was performed at a dilution 1:2000 for 1 h at room temperature. The signal was detected using the SuperSignal West Pico Chemiluminescent Substrate kit (Thermo Scientific, Rockford, IL, USA) in a ChemiDoc transilluminator with Image Lab v5.2 software (both from Bio-Rad Laboratories, Hercules, CA, USA). To check the amount of loaded protein, membranes were incubated with the anti-α-tubulin (T6074, Sigma-Aldrich, St Louis, MO, USA) or anti-GAPDH (6004, Proteintech group, Chicago, USA) antibodies at a 1:10,000 dilution for 20 min, and with the secondary anti-mouse antibody (Bio-Rad Laboratories, Hercules, CA, USA) at 1:2000 for 20 min. Finally, the intensity of the bands was quantified by densitometric analysis using the same software.

### 4.9. Cell Proliferation

To evaluate *ADAM12*’s role in TNBC cell proliferation, BT-549 and Hs578T cells transfected with the scramble and two shADAM12 were seeded (1 × 10^4^ cells/well) and monitored for 6 days by real-time cell analysis (iCELLigence system, ACEA Biosciences, San Diego, CA, USA), as previously described [58].

### 4.10. Cell Migration

To explore the effect of *ADAM12* silencing on BT-549 cell migration, cells transfected with the scramble, shADAM12_1 and shADAM12_2 were seeded into six-well plates at a density of 2 × 10^5^ cells per well. When they reached 70% confluence, cells were serum-starved for 8 h and three scratches were made in the cell monolayer with a 10 μL pipette tip. Cells were washed with phosphate-buffered saline 1× and maintained in culture medium containing 5% FBS. After 24 h, 10 pictures at 50× magnification were taken with a Leica DMLI LED microscope (Leica Microsystems, Wetzlar, Germany) and the mean scratch width was determined using NIS-Elements 4.3 software (Nikon, Melville, NY, USA) from at least 10 measurements taken from each picture.

### 4.11. Drug Response

To determine whether the *ADAM12* gene was involved in any response to chemotherapy, the sensitivity of TNBC cell lines to doxorubicin and paclitaxel (both from Selleck Chemicals, Houston, TX, USA) was first evaluated. For dose–response curves, 1 × 10^4^ cells/well were plated in 100 μL of culture medium in 96-well plates, allowed to attach overnight, then treated with a wide range of doxorubicin or paclitaxel doses for 72 h, using DMSO as a vehicle control (Sigma-Aldrich, St Louis, MO, USA). Cells were fixed and stained with a paraformaldehyde-containing crystal violet solution (Sigma-Aldrich, St Louis, MO, USA). After washing, dead cells were removed, and cell viability was estimated by measuring the optical density of the remaining living cells at 590 nm. The IC_50_ values for each drug in each cell line were calculated using GraphPad Prism v5.1 (GraphPad Software, San Diego, California, USA) by fitting data to a sigmoidal curve. Finally, BT-549 cells transfected with scramble, shADAM12_1 and shADAM12_2 were treated with the IC_50_ value of the drug, and cell viability was measured at 72 h, as described above.

### 4.12. Statistical Analysis

Demographic, clinical and pathological data were summarised as frequencies (and percentages) or means/medians (and ranges), as appropriate. Medians of methylation and immunohistochemical expression in tumour, adjacent-to-tumour and non-neoplastic tissues were compared using the Mann–Whitney U test. Differences between proportion of hypermethylated and hypomethylated cases were calculated with Fishers’ exact test. The effects of *ADAM12* silencing on cell proliferation, migration and drug response were compared in scramble-, shADAM12_1- and shADAM12_2-transfected cells using Student’s two-tailed unpaired samples t-test. Finally, Kaplan–Meier plots and Gehan–Breslow–Wilcoxon tests were used to examine the association of *VWCE, TSPAN9* and *ADAM12* methylation status or protein levels with progression-free survival (PFS) and overall survival (OS). A multivariate Cox regression model was fitted to test the independent contribution of each variable to patient outcome. Hazard ratios and 95% confidence intervals were used to estimate the effect of each variable on the outcome. The association between *ADAM12* methylation and age was estimated by the Mann–Whitney U test and the association between *ADAM12* methylation and stage was estimated by Fisher’s exact test.

## Supplementary Materials

The following are available online at https://www.mdpi.com/1422-0067/21/3/903/s1.
