# Supplementary material for: ADAM12 is A Potential Therapeutic Target Regulated by Hypomethylation in Triple-Negative Breast Cancer
Source: Int J Mol Sci. 2020 Jan 30;21(3):903. doi: 10.3390/ijms21030903 (PMC7036924; doi:10.3390/ijms21030903)
Supplement: Supplementary file 1 [file ijms-21-00903-s001.pdf]

SUPPLEMENTARY INFORMATION

The supplementary information consists of four tables and three figures.

Supplementary Tables

Supplementary Table S1. Methylation status of *VWCE*, *TSPAN9* and *ADAM12* genes in breast samples. Median and range of methylation percentage was measured by pyrosequencing in 24 non-neoplastic breast tissues (N), 50 TNBCs (T), and paired adjacent non-neoplastic tumour tissues (A). Methylation levels of the CpG included in the array (*) and contiguous CpGs for each gene are shown.

	VWCE				*TSPAN9*					*ADAM12*		
	CpG1	CpG2*	CpG3	Mean of CpGs	CpG1	CpG2	CpG3	CpG4*	Mean of CpGs	CpG1*	CpG2	Mean of CpGs
**N**	32 (19-49)	32 (21-53)	24 (18-78)	29 (29-59)	21 (5-40)	9 (1-24)	21 (3-39)	8 (2-20)	18 (3-32)	26 (15-77)	26 (16-76)	25 (15-77)
**T**	6 (0-50)	6 (0-49)	3 (0-100)	7 (0-49)	1 (0-2)	2 (1-4)	0 (0-2)	0 (0-1)	1 (1-81)	9 (2-21)	8 (2-14)	10 (2-22)
**A**	20 (0-73)	49 (0-85)	31 (0-98)	36 (0-70)	1 (0-2)	2 (2-53)	0 (0-27)	0 (0-0)	1 (1-35)	9 (0-26)	1 (0-20)	9 (1-26)

Supplementary Table S2: Cox regression model shows the independent effect of each prognostic factor on progression-free survival (HR, Hazard ratio, CI, confidence interval).

**Variable**	***p*-value**	**HR**	**95% CI (HR)**
Age	0.71	1.00	0.30-7.60
Stage	0.04	0.25	0.07-094
ADAM12 hypomethylation	0.24	2.13	0.60-7.60

Supplementary Table S3. Pathological and clinical characteristics of the TNBC patient series.

Feature	Frequency (%)
**Histological grade**	
1	0/50 (0)
2	4/50 (8)
3	46/50 (92)
**Lymph node involvement**	
Yes	20/50 (40)
No	30/50 (60)
**Stage**	
I	17/50 (34)
IIA	19/50 (38)
IIB	8/50 (16)
IIIA	6/50 (12)
**Age** (years)	Mean 58.8<br>Range 31-89
**Tumour size** (cm)	Mean 2.1<br>Range 0.9-5
**Progression-free survival** (months)	Mean 79.4<br>Range 2-172
Yes	13/50 (26)
No	37/50 (74)
**Overall survival** (months)	Mean 87.1<br>Range 10-172
Exitus	12/50 (24)
**Chemotherapy**	
Yes	45/50 (90)
No	5/50 (10)
**Hormone therapy**	
Yes	0/50 (0)
No	50/50 (100)
**Radio therapy**	
Yes	41/50 (82)
No	9/50 (18)

Supplementary Table S4. Primer sequences used in PCR and pyrosequencing, resulting amplicon size and specific melting temperature (Tm). Primers were designed using PyroMark Assay Design 2.0 software (Qiagen, Hilden, Germany).

Gene	Forward primer	Reverse primer	Sequencing primer	Amplicon size (bp)	Tm (ºC)
ADAM12	TATTAGTTAGTTTTTGGGTTTGTAGT	[Btn]ACACCATCCAACTTTTCAAACTAAAACT	AACTAAAAACCATAACTCTACTACT	108	54,5
TSPAN9	[Btn]AGAGGGGGAGTGTAAGGTT	ACTTAACAAAATCCCAATCCTTACTATCCA	CCTTACTATCCAAAAATAAACTC	110	59
VWCE	GGGTTTTATAGATAGGGGTTATGTT	[Btn]CTCCACCCACACCCCCTACC	GTTTTGTTTTCGAAGTTTGTTTTTT	155	61,8

Supplementary Figures

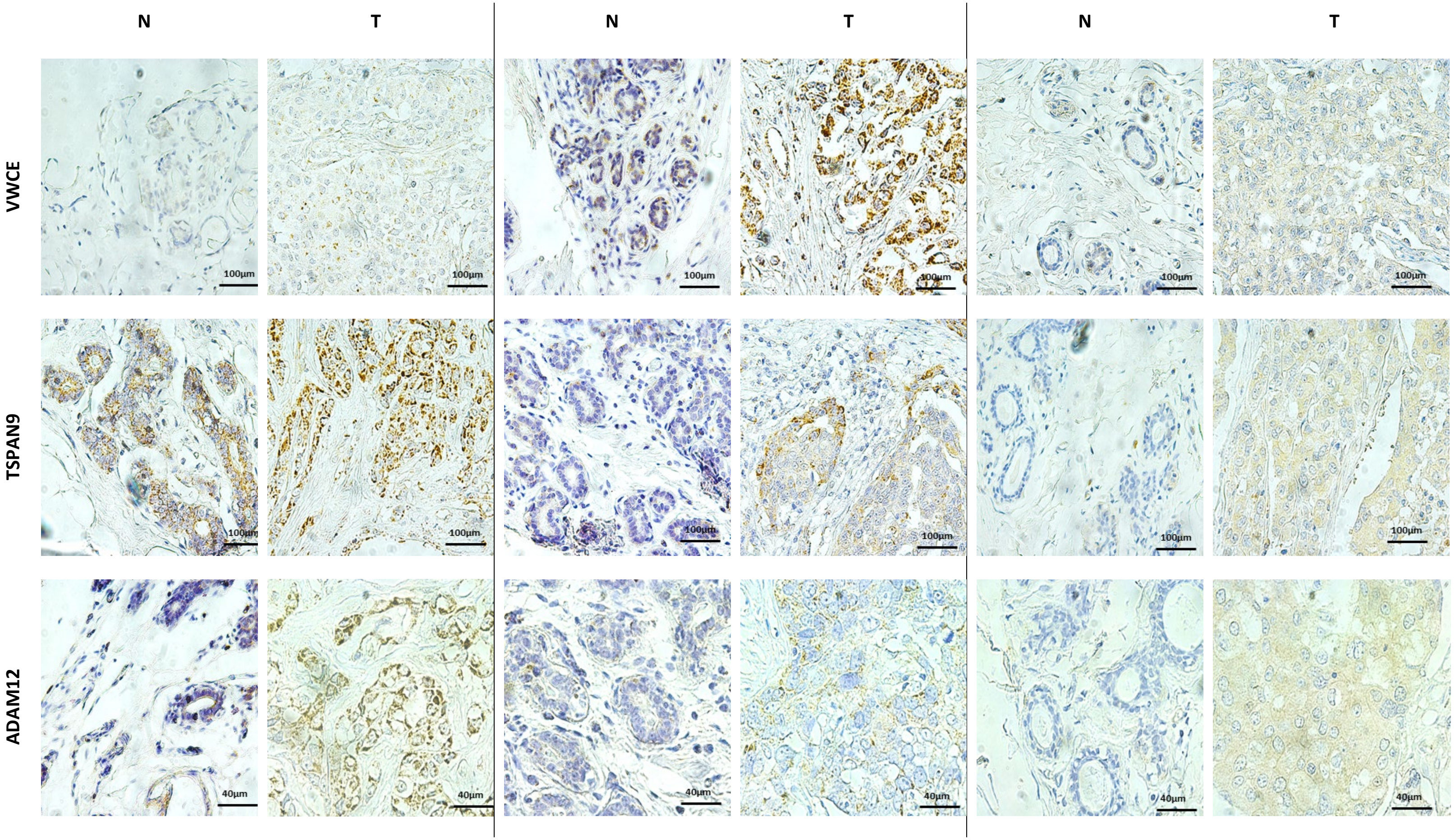

Supplementary Figure S1. Representative IHC of non-neoplastic (N) and triple-negative breast cancer (T) tissues of VWCE, TSPAN9 and ADAM12 proteins. Three representative images are shown. Images from first and second row were acquired at 400X magnification, and images from last row at 630X magnification

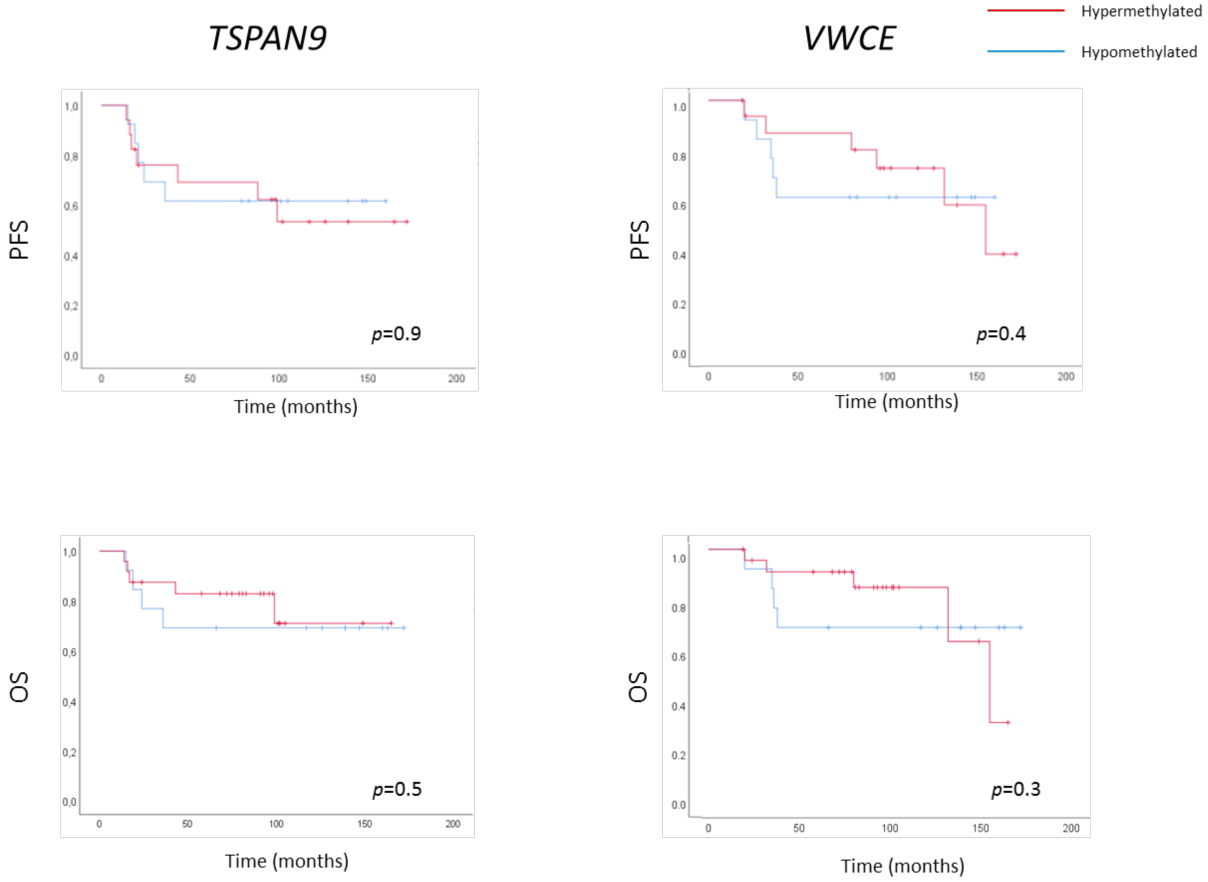

Supplementary Figure S2. Clinical value of *TSPAN9* and *VWCE* hypomethylation in TNBC**.** Association between methylation and progression-free survival (PFS) (up) and overall survival (OS) (down) in our series of TNBC patients.

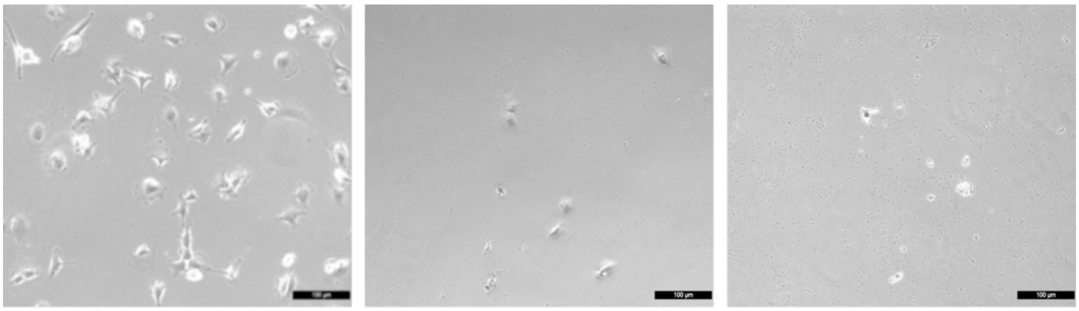

scr sh1 sh2

Supplementary Figure S3. *ADAM12* silencing in Hs 578T cells. Hs 578T cells were transfected with pHIV1-SIREN+scramble (scr), pHIV1-SIREN+shADAM12_1 (sh1), and pHIV1-SIREN+shADAM12_2 (sh2), and selected with puromycin for 2 weeks. Images were acquired at 100X magnification.
